# Analysis of the Temperature Distribution on the Surface of Saddle-Shaped Briquettes Consolidated in the Roller Press

**DOI:** 10.3390/ma14071770

**Published:** 2021-04-03

**Authors:** Michał Bembenek, Andrzej Uhryński

**Affiliations:** Faculty of Mechanical Engineering and Robotics, AGH University of Science and Technology, A. Mickiewicza 30, 30-059 Krakow, Poland; uhrynski@agh.edu.pl

**Keywords:** briquetting, roller press, temperature distribution, thermography, briquetting pressure

## Abstract

The unit pressure in the fine-grained material consolidation process in the roller press can reach over hundred MPa and is a parameter which results, among other things, from the properties of the consolidated material and the compaction unit geometry. Its value changes depending on the place on the molding surface. Generating different pressure on the surface of briquettes makes their compaction different. One’s own and other researchers’ experience shows that, in the case of exerting high pressure on the merged fine-grained material, the higher unit pressure exerted on the material, the higher temperature of the consolidated material is. The temperature distribution on the surface of the briquettes can testify the locally exerted pressure on the briquette. The stress distribution in the briquetting material is still a subject of research. The article includes thermography studies of the briquetting process of four material mixtures. Thermal images of briquettes were taken immediately after they left the compaction zone as well as forming rollers. The obtained thermograms and temperature variability at characteristic points of the surface of briquettes were analyzed. The correlation between the temperature distribution and the stress distribution in the briquettes was determined.

## 1. Introduction

Thermography is a non-destructive, non-contact method of detecting, registering, processing, and then visualizing infrared radiation. The obtained thermogram is a representation of the temperature distribution on the surface of the observed object. The non-contact and non-invasive measurement of the tested objects in most applications is the basic criterion for the use of thermal imaging techniques.

One of the first fields that successfully used thermography has been construction in its broadest sense. It is used to analyze the thermal tightness of buildings, defects in the heating system [1], as well as to identify areas which are at risk of moisture condensation and mold [2]. This technique (due to its non-invasiveness) has also been well received in medicine and scientific research related to it [3,4,5]. Thermography is also used in the machining of materials. This applies not only to the control of the workpiece temperature [6], but also to the general control of the temperature increases and distribution in the machining zone [7], or the chip surface temperature [8]. It has also been mentioned that the use of thermography occurs in the diagnosis of the welding process and the assessment of the quality of these joints [9,10]. Thermography is used in the widely understood diagnostics of machines and their components, starting from CNC (Computer Numerical Control) machine tools [11], through mechanical expanders [12], rotating machines [13], belt conveyors [14], internal combustion engines (catalytic reactors) [15,16], bearings and bearing nodes [17,18], brakes [19], up to electrical devices and machines [20,21,22,23]. Attempts have been made to correlate strength tests and stresses in the material with the amount of heat generated [24]. Loaded ropes and steel chains were examined in order to determine their weak points [25]. Plastic strains and cavitation of polypropylene under tension were analyzed [26], and a system for comprehensive thermomechanical analysis of materials was suggested [27]. Strength tests were also performed during a bending test of thin-walled composite beams made of matrix composites [28]. A methodology for the use of thermal imaging techniques to assess the extent of fatigue degradation of polymeric materials (in particular epoxy composite) was also developed [29]. In addition to passive thermography, active thermography is also known. It is a method of stimulating the object of research, for example, by providing a thermal impulse [30] or inducing this effect by means of acoustic activation [31], including ultrasound [32] or microwaves [33], and then observing the path of its propagation by a thermal imaging camera in the facility. This type of thermography is especially used in defectoscopy [34,35].

Thermal phenomena are a rich source of information about technological processes and changes or irregularities occurring during them [36], which allows for the control of process parameters and the necessary modification. Thermography was used, among others, to control the stability of the extrusion process of poly (vinyl chloride) [37], textiles [38,39,40], tire vulcanization [41], production of casting molds [42], the process of casting and cooling steel [43] iron ores (with biomass) [44], the quality of moldings made of loose materials (microcrystalline cellulose) leaving the compaction zone [45], and even in the process of cooling of natural rock materials [46]. Another field of application for thermography has become research on materials and their structure [45,47]. The possibilities of using the thermography method in the analysis of the degree of destruction of metal-fiber laminates subjected to dynamic impacts at low speed were described [48]. A prototype of a thermographic apparatus for non-destructive testing of composites was also developed [49].

**Figure 1:**
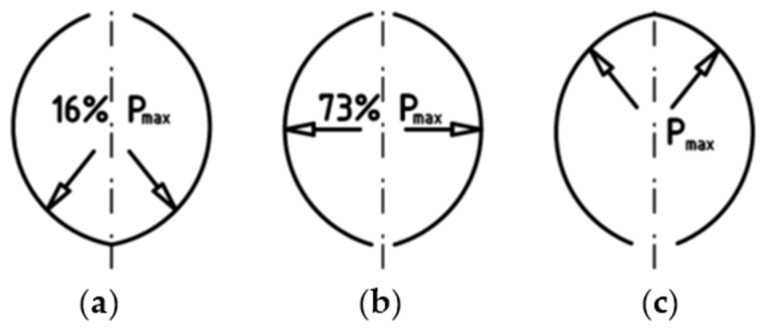
Unit pressure value distribution during the briquetting process in a roller press. P_max_, maximum unit pressure [64]: (**a**–**c**) the successive compaction phases during the rotation of the rollers.

Despite such a wide application of thermographic research, it is very rarely used to study agglomeration processes [45]. The pressurized agglomeration is the key process that is presently employed in the power [50,51,52,53,54], heavy [55,56,57,58], chemical [59,60], and pharmaceutical industry [61,62,63]. Industries have willingly used roller presses rather than other briquetting machines, for example, screw or punch briquette machines [50,53], due to constant operability with a relatively low demand for energy [64,65]. A standard roller press design usually contains the following subassemblies: drive system, working rollers cage, roller support system, and compaction unit. The compaction unit is a particularly important unit among the systems for pressurized agglomeration [54,61,66]. A very important element of a properly conducted agglomeration is effective preparation of the material and its dosing [67,68]. The briquette forming process takes place between two rollers, which rotate in mutually opposite directions and on the surfaces of which there are molding cavities distributed in a proper way [69]. In traditional roller presses, working surfaces of rollers are provided with cavities which are arranged so as to be their mutual mirror images on both rollers [64]. The compaction unit of this type is referred to in the literary sources as symmetrical [64,65]. Mutual differentiation of the working surfaces of both rolls prevents unfavorable phenomena from occurring during material consolidation [55]. In this way, an asymmetric compaction unit is established. The briquettes manufactured in a traditional asymmetrical compaction unit are in the shape of a saddle. This is particularly useful for materials that are difficult to briquette in a roller press, i.e., those that are characterized by a high moisture, high compaction degree necessary for consolidation, low bulk density, the presence of hydrophobic grains, those that tend to be suspended in hoppers and dispensers, and those with a high elastic deflection after pressure is removed. The use of an asymmetric compaction unit enables an increase in the moisture range within which the material can be briquetted [64,65]. It also eliminates briquettes from cracking in half along the plane of mutual closure of cavities on both rolls [55,64]. The unit pressure in the fine-grained material consolidation process often reaches several hundred MPa and is a parameter which results, among other things, from the properties of the material being consolidated and the compaction system geometry [54,61,70]. Its value changes depending on the place on the molding surface [71,72]. This is one of the reasons that the briquette compaction is not uniform throughout its volume, as shown by the research presented in the works [70,73]. It was also proved in simulation studies using the Finite Element Method [74]. In the case of the gravity feeder for the symmetrical compaction unit, the lowest unit pressure value occurs in the lower area of the cavity (Figure 1). A similar stress distribution can be expected for an asymmetric compaction system; however, such tests were not carried out with gravity feeding.

Our own experiments carried out during the determination of the compaction level, characteristics of fine-grained materials [69,75] performed in a closed die, show that the compacted material heats up when high pressure is exerted on it. This is also confirmed by the experiments done by Litstera et al. [45]. Based on the microcrystalline cellulose, they proved the temperature distribution in a roller compaction process (briquetting with a flat rollers) across the width of the ribbon is not uniform. There are temperature differences between the central area and the edges of the ribbon. It is related to the inter alia with a different degree of material compaction (higher density in the middle of the part), which is caused by uneven material flow in the press feeder. During consolidation, due to inner friction and work, heat is generated between the particles of the merged material which causes a temperature change in a certain volume of the briquette. The higher unit pressures exerted on the material, the higher the temperature of the consolidated material is. The temperature distribution on the surface of the briquettes can also be related to locally exerted pressure on the briquette. The stress distribution in the briquetting material is still the subject of research. Therefore, the IR thermography studies of the briquetting process were conducted in order to verify the use of thermography to study the course of the briquetting process in a roller press.

## 2. Materials and Methods

**Figure 2:**
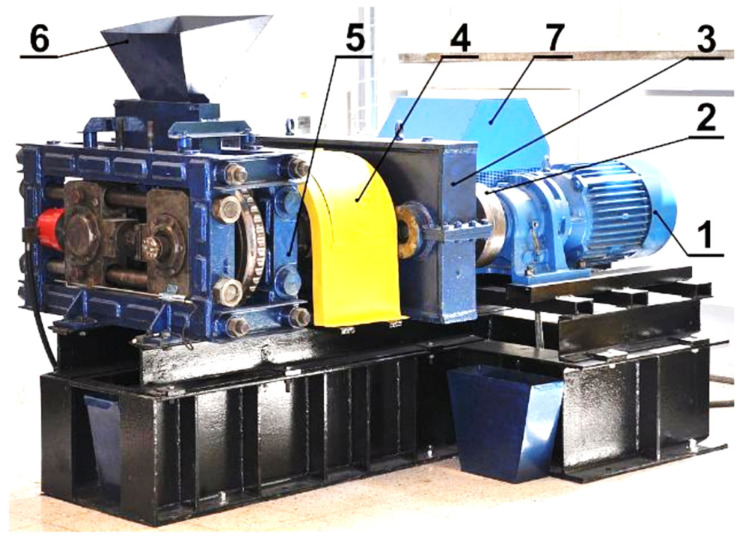
LPW 450 laboratory roller press: (**1**) gear motor with a cycloidal transmission; (**2**) flexible clutch; (**3**) gearbox; (**4**) enclosure of Oldham couplings and friction clutch; (**5**) molding rollers cage; (**6**) gravity feeder; (**7**) hydraulic system of sliding roller support [69].

**Figure 3:**
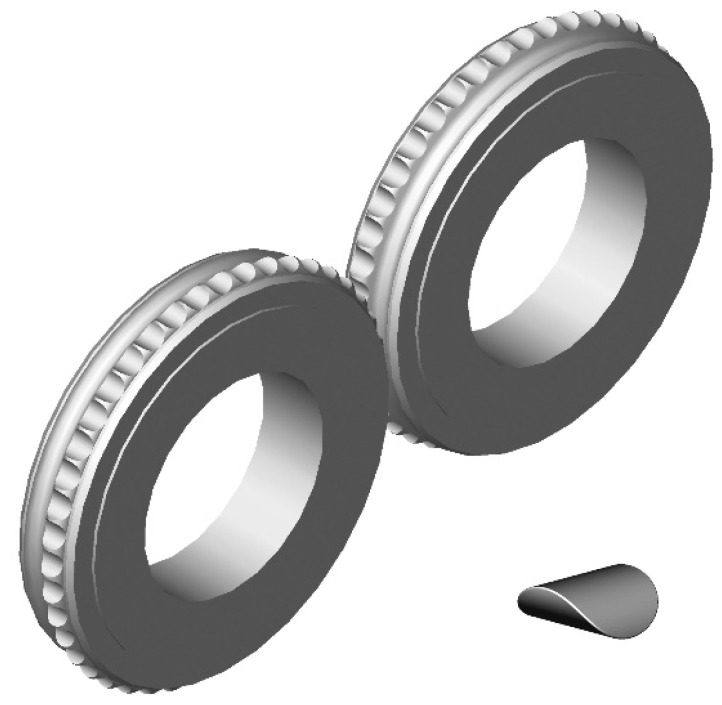
Molding rings used in roller press compaction unit with a saddle-shaped briquette [69].

**Figure 4:**
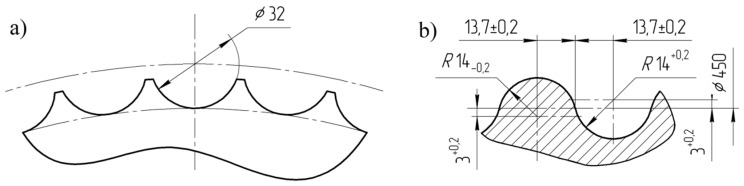
Geometry of molding cavities on the working surface of rolls used for tests: (**a**) front view and (**b**) cross section through the groove [69].

The thermographic research of briquetting process in the roller press was done using a roller press (AGH University of Science and Technology, Krakow, Poland) (Figure 2) with a 450 mm roller pitch diameter with an installed compaction unit for production of the saddle-shaped briquettes with a size of 31 × 30 × 13 mm and a rated capacity of 6.5 cm^3^ (Figure 3). The outline view of the molding surface used to consolidate the material is presented in Figure 4. The press was equipped with a cycloidal gear motor with a power of 22 kW and a frequency converter that enabled infinitely variable control of the revolutionary speed of the rolls. All materials were briquetted using a gravity feeder with a roller revolutionary speed of 0.85 RPM, which corresponded to the peripheral speed of the rolls equal to 0.02 m/s with an inter-roll gap of 1 mm, which caused each briquette in the forming row to fall out approximately 1.6 s.

Materials of three various origins and various chemical compositions and structures were selected for the tests:

- material of inorganic origin—hydrated lime,
- material of organic origin/fuels: charcoal,
- heavy industry waste: electric arc furnace dust (EAFD) mixtures.

Before the consolidation process, four mixtures of the materials referred to above were prepared. They were thoroughly mixed and brought to a proper moisture, enabling them to be consolidated in a roller press. Binders were added. Moisture was determined by the weight method at 105 °C until constant weight was obtained. The Vibra AJH 420 CE (Tokyo, Japan) scale was used.

**Mixture 1. (M1)**

**Figure 5:**
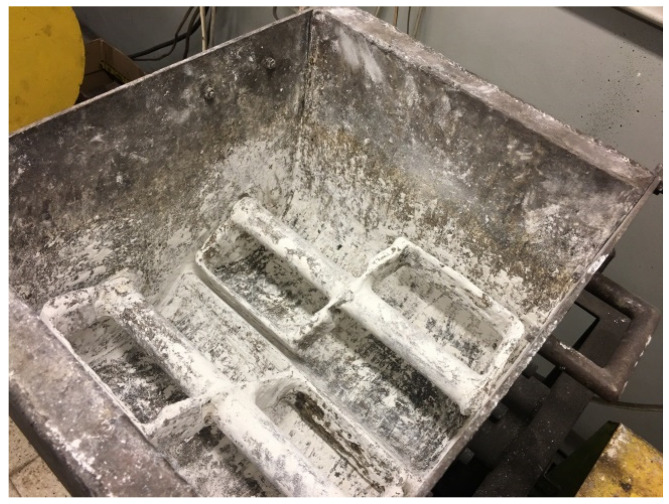
The view of Z-blade mixer used in the test for material preparation.

The mixture contained 47.7% of EAFD, 36.7% of scale, 7.3% fine coke breeze, 5.5% 80° Bx molasses, and 2.8% calcium hydroxide. The last two ingredients acted as a binder [7]. The mixture was mixed approximately 10 min in the double-arm Z-blade mixer with four rectangular mixing elements with dimensions 190 × 90 mm and the shaft rotating speed 55 RPM (Figure 5). Its moisture content was 4.6%.

**Mixture 2. (M2)**

The charcoal fines with a grain size down to 3 mm were mixed in the Z-blade mixer for approximately 10 min. with a 4% of starch. The moisture content of the mixture was 26.9%.

**Mixture 3. (M3)**

The pre-compacted charcoal fines were mixed with a 4% of starch. The moisture content of the mixture was 26.9%. The pre-compacted process involves briquetting and crushing the consolidated briquettes to a size below 10 mm.

**Mixture 4. (M4)**

Its composition was 83.3% calcium hydroxide manufactured by Lhoist (EN 459-1 CL 90-S) (Lhoist, Limelette, Belgium) and 16.7% water. The mixture was mixed in Z-blade mixer for about 30 min. The moisture content of the mixture was 16.9%.

**Figure 6:**
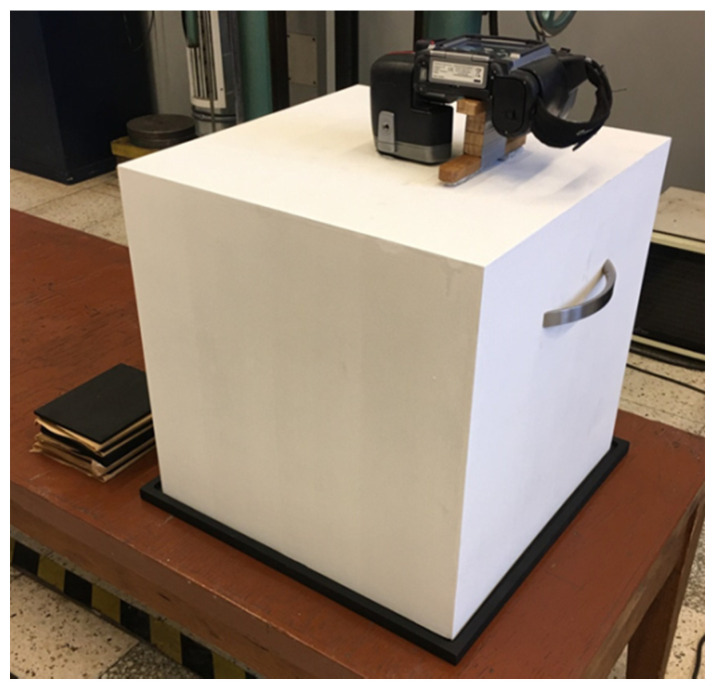
The view of the light-tight imaging station for taking photos during measurements, eliminating the effects of external radiation.

**Figure 7:**
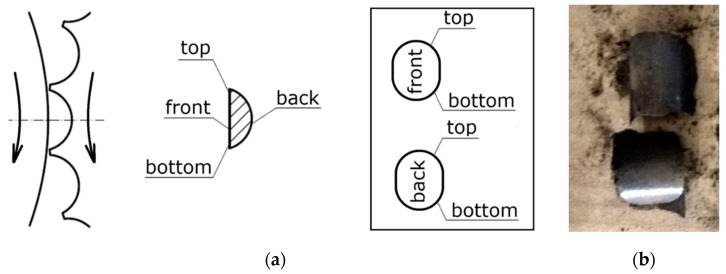
The briquettes arrangement: (**a**) in the compacting unit, (**b**) on the cardboard pad.

The FLIR T335 Thermal Imaging Camera (Wilsonville, OR, USA) was used for the tests. The operating temperature range of the camera is: −20 to +650 °C, and the temperature measurement error: ± 2 °C or ± 2% of the measurement value. The camera is equipped with a microbolometric matrix with a resolution of 320 × 240 pixels, a 25° × 18.75° lens, and a 0.05 °C Noise Equivalent Temperature Difference sensitivity. Before the tests, it was necessary to calibrate the camera. The ambient temperature during tests was 18.6 °C. In the laboratory, access to daylight and artificial light to the measuring station was eliminated. In addition, a light-tight imaging station was made to block the inflow of the light to the working area of the thermal imaging camera (Figure 6), which eliminated the reflection of radiation disturbing the test result and ensuring repeatability of the distance between the camera and the tested briquette. Each time, two briquettes were ejected from the roller press at a time, and were caught and transferred to a cardboard plate in heat-insulating gloves immediately after leaving the compaction unit. Then, the briquettes and the pad were placed in a measuring station and thermal images were taken. The time between catching the briquettes and taking the photo was about 3 s. After each test, the cardboard pad was changed to make the test conditions reproducible as the pads got hotter (the pads got hotter from the briquettes as time went on). Due to the low peripheral speed of the rollers (0.02 m/s), it was possible to transfer the briquette to the pad directly after it was removed from the compaction unit, while maintaining a precise control of briquette top-bottom, front-back orientation. Briquetting tests with the faster peripheral speeds were unsuccessful as it was not possible to catch the briquettes in a controlled and reproducible orientation. The photos of the briquettes were taken in such a way that their “top” (Figure 7) was always located up the top edge of the image. Two briquettes were placed on the pads in two positions: “front” and “back”, as it shown in Figure 4b.

**Figure 8:**
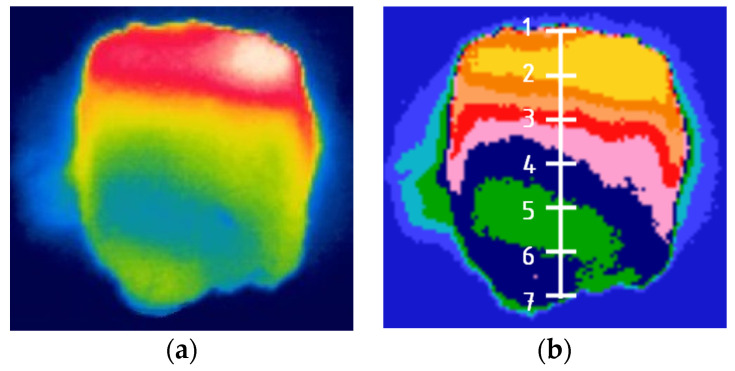
Thermogram: (**a**) unprocessed, (**b**) processed in FLIR Quick Report with measuring points where temperature readings were taken to prepare the temperature distribution on the briquette surfaces.

From 3 to 10 thermal images were taken of the briquettes from each of the mixtures, out of which 3 with the highest maximum temperature were selected for further analysis (Figure 8a). The photos were analyzed using the FLIR Quick Report program v. 1.2 SP1 (Wilsonville, OR, USA). First of all, the maximum temperature was determined for each type of briquette, both at the front and back, and their measurements were averaged.

In order to examine the temperature distribution on the briquette surfaces, into the thermograms, the grid shown in Figure 8a was fitted. The measuring points were spaced 5 mm from each other (Figure 8b). For each thermogram, 7 temperature values were read at points in the vertical axis of the briquette.

The next stage of the research was attempts to determine the temperature distribution on the rollers immediately after the consolidation process. The thermographic images of the rollers were taken with and without an additional anti-reflective coating. The rollers were coated with an anti-glare spray containing talcum powder CRC Crick 130 (CRC Industries Europe, Belgium). The thermographic images of rollers stored in the laboratory at ambient temperature were also taken.

## 3. Results and Discussion

Good quality briquettes which did not crumble were obtained from all the materials used in the tests. In tests where fine-grained charcoal was merged, the shape of the edge of briquettes was more irregular; hence, the images of the briquettes on the thermograms had less-clear edges. However, it did not hinder their thermal imaging analysis.

The first stage of the research was to produce a pilot batch of briquettes from each of the mixtures. The briquettes were then seasoned at ambient temperature for 6 h and then thermal images of all four types of briquettes were taken together. The analysis of the images showed that the briquettes have the same temperature as the ambient temperature. The temperature of all briquettes was the same on the surfaces of each of the briquettes and no differences were noticed in the temperature distribution. For briquettes from each mixture, images were also taken after the next 5, 10, 15 s briquetting in order to analyze the cooling process of the briquettes. Based on the analysis of the briquette cooling gradient on subsequent images, it was found that the time between making the briquettes, their transfer to the station, and taking the first photo did not significantly affect the temperature changes. Then the actual tests began.

**Figure 9:**
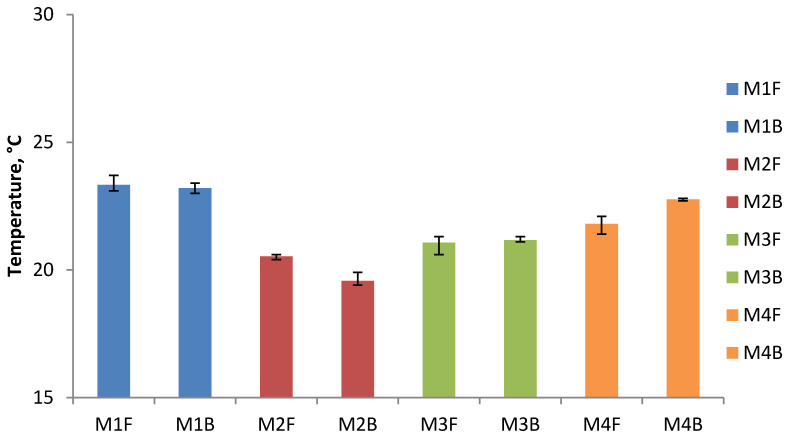
The results of average minimum temperature measurements on the surface of briquettes after briquetting: F—front, B—back.

**Figure 10:**
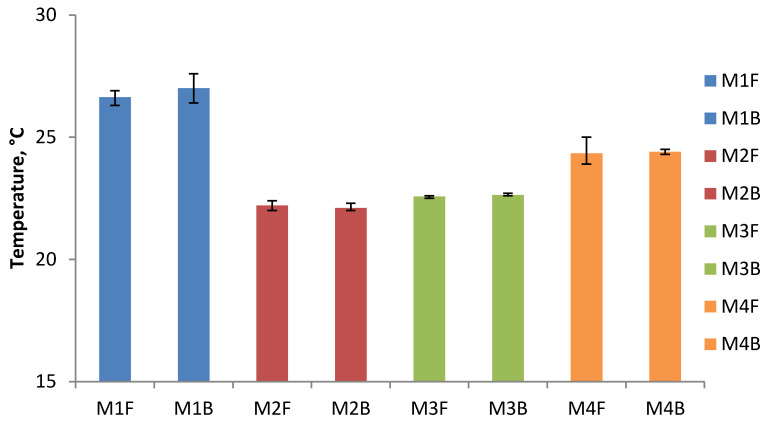
The results of average maximum temperature measurements on the surface of briquettes after briquetting: F—front, B—back.

The results of the minimum and maximum temperature for each type of briquettes on the front and back sides and their average values are presented in Table 1 and Table 2 and in the Figure 9 and Figure 10.

The summary graph of the minimum surface temperatures (Figure 9) shows that the lowest temperature, both on the front and back surfaces, is obtained for the briquettes made of Mixture 2 (charcoal fines mixed with a 4% of starch without pre-compaction). The minimum temperature of these briquettes is 1.0 °C higher than the ambient temperature.

The summary graph of the maximum surface temperatures (Figure 10) shows that the highest temperature, both on the front and back surfaces, is obtained for the briquettes made of Mixture 1 (47.7% of EAFD, 36.7% of scale, 7.3% fine coke breeze, 5.5% 80° Bx molasses, and 2.8% calcium hydroxide). The maximum temperature of these briquettes is 9.4 °C higher than the ambient temperature. The briquettes made of calcium hydroxide are also characterized by a similar maximum temperature on the surface. The highest temperature on the surface of briquettes made of fine charcoal (M2 and M3) is lower than both of the above samples (M1 and M4). It amounts to 22.2 °C for non-compacted briquettes and 22.6 °C for compacted briquettes, respectively. The difference of 0.4 °C between the two samples can be explained by the heating of Mixture 3 during its pre-compaction. The maximum temperatures on both sides of the briquette are very similar to each other. The biggest difference between the front side and the back side was recorded for Mixture 1 and it amounts to 0.4 °C. The differences between the maximum and minimum temperatures measured on the briquette surface are 3.8 °C for the Mixture 1, 2.5 °C for the Mixture 2, 1.5 °C for the Mixture 3, and 2.5 °C for the Mixture 4.

**Figure 11:**
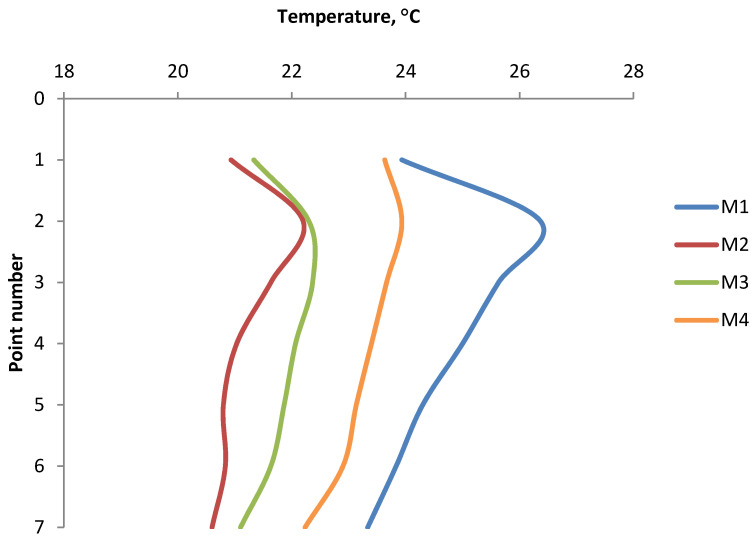
The temperature distribution on the front side briquette surfaces.

**Figure 12:**
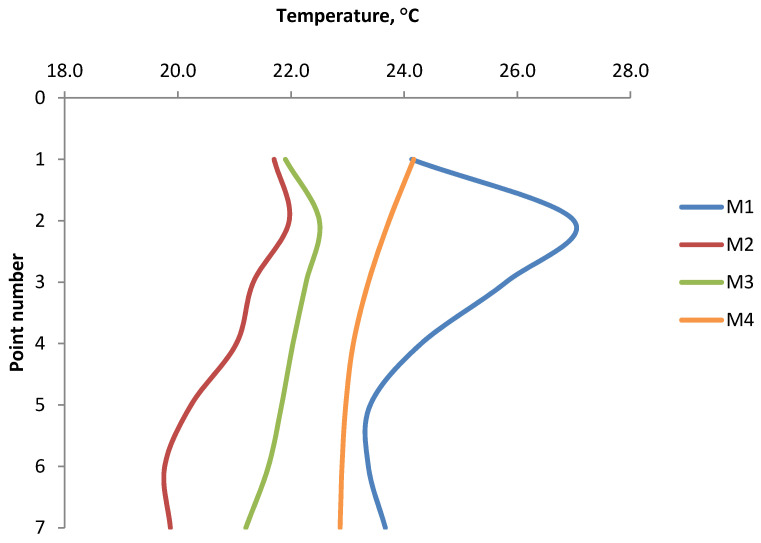
The temperature distribution on the back side briquette surfaces.

The results of the temperature distribution on the briquette surfaces for each type of briquette on the front and the back sides and its average value are presented in Table 3 and Table 4 and in the Figure 11 and Figure 12.

Analyzing the graphs of temperature distribution on both the front and back surfaces of the briquettes (Figure 11 and Figure 12), it can be concluded that the characteristics of the temperature variation curve are similar for all the tests. The temperature values increase from point 1 to the maximum value in point 2 or 3, and then decrease to point number 7. The maximum temperature values in all cases were obtained in point 2, except for the temperature distribution on the front part of the Mixture 3 briquette, where the highest temperature was recorded in point 3. The conclusion is that the areas with maximum temperature are in the upper and middle upper parts of the briquette. In the temperature distribution in the front side of the briquettes, the temperature values at measuring points 1 and 7 are similar to each other. These points lie at the border edges of the briquette. On the back sides of the briquettes, the temperatures in point 1 are slightly higher than in point 7. The lowest temperatures at all the measurement points were recorded on the surfaces of briquettes made of a mixture of charcoal without pre-compaction (M2). Despite the smaller range of temperature values, the thermograms clearly show that the shape of the temperature gradients is analogous to the thermograms obtained in other mixtures. Tests carried out on Mixture 2 and Mixture 3 (the same mixture of charcoal, only prepared differently for consolidation) show that the temperature distribution on the surface of briquettes depends primarily on the geometry of the compaction unit and on the preparation of the material.

**Figure 13:**
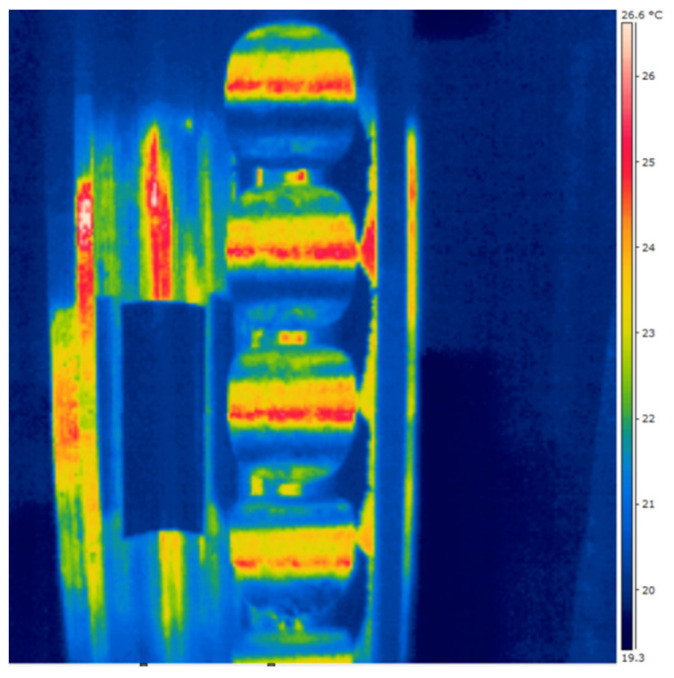
Working roller thermogram with reflections of radiation before briquetting process.

The first tests to take the thermographic images of the working rollers before the briquetting process are shown in Figure 13. In that case, the reflection of radiation was recorded, and therefore, they were considered unsuccessful. This is also confirmed by the control tape stuck to the roller which was not reflected. The next step was to eliminate all reflections. The thermogram of the roller after briquetting clearly shows that its surface was heated up during the process and the temperature distribution was not uniform. Despite eliminated access to daylight and artificial light in the laboratory, the reflection was still visible. The control tape after the briquetting was destroyed due to very high pressure on the rollers surface.

Then, thermal images of the rollers stored and not used in the laboratory at ambient temperature were taken. Despite eliminated access to daylight and artificial light in the laboratory, the reflections were still visible. Despite their constant temperature, the thermograms showed variation in their surface temperature by about 1 °C, which made the accuracy of the measurements too low for the correct analysis of the results. In subsequent tests, the rolls were coated with an anti-reflex spray CRC Crick 130 (CRC Industries Europe, Zele, Belgium). The attempts were also considered unsuccessful. The spraying of the aerosol after the briquetting process cooled the surface of the rollers and made it impossible to record the actual temperature gradients. The coating applied before consolidation, after the briquetting process, was destroyed. Despite the observance of the strict test conditions, attempts to measure the temperature distribution on the surface of working rollers did not bring positive results.

## 4. Conclusions

The conducted research allows to conclude that a thermograph is an interesting research method in the context of using it to analyze the processes of briquetting fine-grained materials. At the same time, this method requires very good preparation of the test stand in order to eliminate the influence of undesirable external factors, like radiation sources that may prevent the reliable processing of the obtained results. The attempts to measure the temperature distribution on the surface of working rollers did not bring positive results. Despite the observance of the strict test conditions, the external disturbances on the roller surface could not be eliminated. For each type of the merged material, the temperature in the upper part of the briquette was the highest. The obtained temperature distributions coincide with the bulk material deformation in the forming cavity during briquetting process in asymmetrical compaction unit presented in the work [70]. From the analysis of both distributions, it can be concluded that higher local briquette temperatures in a given place correspond to better local density. Therefore, in the case of saddle-shaped briquette in the upper part of the briquette, the stresses and strain are greater, and those in the lower part—smaller, which is also confirmed by the recent research presented in the paper [76].

The study of the temperature distribution presented in the paper can also be related to the actual types of pressure exerted on the bottom (central part) of the forming cavity presented in the literature. They amount to approximately 65 MPa for the M1 mixture [69], while for the M4 mixture it is approximately 30 MPa [77]. Different pressure generated on both materials also correlates with the temperatures obtained on the briquette surface in their central part. For mixture M1, the measured temperature at the center of the forming cavity (point 4) is higher, and so is the pressure. It should be noted that each material may have a different temperature response to pressure. This was proved by the own research of the pressure exerted on M2 and M3 materials during briquetting in the roller press. The pressures measured for these materials are 45 and 50 MPa, respectively. This corresponds to the difference of measured temperatures on the briquette surfaces of M2 and M3 materials (higher pressure, higher temperature). However, it was proved that materials do not directly correlate with pressures and temperatures for each other. On the basis of thermography studies, it is concluded that measuring the pressure in the central part is not the maximum pressure exerted on the briquette during forming; however, from the point of view of measurement technology, a different arrangement of the pressure sensor is difficult to implement in technical conditions.

It can be concluded that testing the temperature in the briquette can be an indirect method of testing its degree of compaction and the pressure generated on its surface during briquetting. This method can be widely used to select the right forming volume and geometry to obtain properly compacted briquettes. This method can also be very useful for examining the correction of the shape of forming cavities, because on its basis, it can be seen which part of briquette is not compacted properly, thus which area of the surface forming the cavities should be corrected because in this place, the local temperature will be lower than in other places [69].

## Figures and Tables

**Table 1:** The results of minimum temperature measurements on the surface of briquettes after briquetting.

Side	M1, °C		M2, °C		M3, °C		M4, °C	
	23.1		20.4		20.6		21.4	
Front	23.2	23.3	20.6	20.5	21.3	21.1	21.9	21.8
	23.7		20.6		21.3		22.1	
	23.0		19.4		21.1		22.7	
Back	23.2	23.2	19.4	19.6	21.1	21.2	22.8	22.8
	23.4		19.9		21.3		22.8	

**Table 2:** The results of maximum temperature measurements on the surface of briquettes after briquetting.

Side	M1, °C		M2, °C		M3, °C		M4, °C	
	26.9		22.4		22.6		23.9	
Front	26.7	26.6	22.2	22.2	22.6	22.6	24.1	24.3
	26.3		22.0		22.5		25.0	
	27.6		22.3		22.7		24.3	
Back	27.0	27.0	22.0	22.1	22.6	22.6	24.4	24.4
	26.4		22.0		22.6		24.5	

**Table 3:** The temperature distribution on the front side briquette surfaces in the characteristic points.

Point	M1, °C		M2, °C		M3, °C		M4, °C	
	23.9		20.6		21.3		23.7	
1	23.8	23.9	20.8	20.9	21.3	21.3	23.5	23.6
	24.1		21.4		21.4		23.7	
	25.5		22.2		22.3		24.0	
2	26.7	26.4	22.0	22.2	22.0	22.3	23.8	23.9
	26.9		22.4		22.6		24.0	
	24.9		21.6		22.6		23.8	
3	26.3	25.6	21.3	21.6	22.0	22.4	23.4	23.7
	25.7		22.0		22.5		23.8	
	24.4		21.2		22.4		23.5	
4	25.6	25.0	20.8	21.0	21.7	22.1	23.2	23.4
	25.0		21.1		22.1		23.5	
	24.0		21.1		22.1		23.3	
5	24.7	24.3	20.4	20.8	21.5	21.9	23.0	23.1
	24.2		20.9		22.0		23.1	
	23.9		20.7		21.8		23.0	
6	23.9	23.8	21.1	20.8	21.4	21.6	22.8	22.9
	23.7		20.7		21.7		22.9	
	23.7		20.6		21.4		22.3	
7	23.1	23.3	20.6	20.6	20.6	21.1	22.0	22.2
	23.2		20.6		21.3		22.4	

**Table 4:** The temperature distribution on the back side briquette surfaces in the characteristic points.

Point	M1, °C		M2, °C		M3, °C		M4, °C	
	23.9		21.2		21.9		24.2	
1	24.2	24.1	22.0	21.7	21.9	21.9	24.1	24.2
	24.3		21.9		21.9		24.2	
	27.6		22.3		22.6		23.7	
2	27.0	27.0	21.6	22.0	22.2	22.5	23.6	23.7
	26.4		22.0		22.7		23.9	
	25.9		21.4		22.6		23.5	
3	25.6	25.8	21.5	21.3	22.0	22.3	23.2	23.4
	25.9		21.1		22.2		23.4	
	24.0		21.1		22.2		23.2	
4	24.2	24.3	21.0	21.0	21.9	22.0	22.9	23.1
	24.7		21.0		22.0		23.2	
	23.4		20.3		22.0		23.1	
5	23.2	23.4	20.0	20.2	21.6	21.8	22.8	23.0
	23.6		20.4		21.9		23.0	
	23.0		19.9		21.8		22.9	
6	23.6	23.4	19.4	19.8	21.3	21.6	23.0	22.9
	23.5		20.0		21.7		22.8	
	23.7		20.2		21.4		22.8	
7	23.7	23.7	19.4	19.9	21.1	21.2	23.0	22.9
	23.6		20.0		21.1		22.8	

## Data Availability

The data presented in this study are available on request from the corresponding author.
